# High Expression of *HULC* Is Associated with Poor Prognosis in Osteosarcoma Patients

**DOI:** 10.1371/journal.pone.0156774

**Published:** 2016-06-02

**Authors:** Vanessa Regina Maciel Uzan, André van Helvoort Lengert, Érica Boldrini, Valter Penna, Cristovam Scapulatempo-Neto, Carlos Alberto Scrideli, Alberto Paiva de Moraes Filho, Carlos Eduardo Bezerra Cavalcante, Cleyton Zanardo de Oliveira, Luiz Fernando Lopes, Daniel Onofre Vidal

**Affiliations:** 1 Pediatric Oncology Laboratory, Molecular Oncology Research Center, Barretos Cancer Hospital, Barretos, SP, Brazil; 2 Barretos Children’s Cancer Hospital, Barretos, SP, Brazil; 3 Department of Orthopedic Surgery, Barretos Cancer Hospital, Barretos, SP, Brazil; 4 Department of Pathology, Barretos Cancer Hospital, Barretos, SP, Brazil; 5 Department of Pediatrics, Medicine School of Ribeirao Preto, University of Sao Paulo, Ribeirao Preto, SP, Brazil; 6 Department of Radiology, Barretos Cancer Hospital, Barretos, SP, Brazil; 7 Department of Radiology, Barretos Children’s Cancer Hospital, Barretos, SP, Brazil; 8 Center for Research Support (NAP), Barretos Cancer Hospital, Barretos, SP, Brazil; The Ohio State University, UNITED STATES

## Abstract

Osteosarcoma (OS) is the most common primary bone cancer in childhood. OS is an aggressive disease, and metastatic patients evolve with very poor clinical outcomes. Genetically, OSs are extremely complex tumors, and the related metastatic process is not well understood in terms of the biology of the disease. In this context, long non-coding RNAs (lncRNAs) have emerged as an important class of gene expression regulators that play key roles in the invasion and metastasis of several human tumors. Here, we evaluated the expression of *HULC*, which is an lncRNA that is associated with the tumor metastatic process, and assessed its potential role as a prognostic marker in OS. *HULC* expression was evaluated in primary OS samples using real-time RT-PCR. *HULC* expression status was determined by receiver operating characteristic (ROC) analysis, and its association with survival was assessed using the Kaplan-Meier method. The *HULC* expression level was not significantly associated with the clinicopathological characteristics of the OS patients. However, our data demonstrated that higher levels of expression of *HULC* were associated with lower survival rates in OS patients, both in terms of overall and event-free survival. Elevated *HULC* expression was associated with poor clinical outcomes among the OS patients, which suggests that *HULC* could be a potential prognostic biomarker in OS.

## Introduction

Primary bone tumors represent 5–6% of all tumors of childhood and adolescence (0–19 years), and osteosarcomas (OSs) are the most common primary bone tumor in this age group [1]. Approximately 15–20% of patients with OS present with metastasis at diagnosis as detected by radiological examinations such as computerized tomography (CT). Additionally approximately 40% will progress to metastasis in later stages during treatment [2,3]. Metastases are frequent in the lungs (~ 80%) but can also occur in other sites. Metastatic disease sites commonly exhibit resistance to treatment, which results in a low patient survival rate of approximately 20% over five years [3,4]. In Brazil, patients enrolled in clinical studies III and IV of the Brazilian Osteosarcoma Treatment Group (BOTG) exhibited 50.1% and 39% 5-year overall and event-free survival rates, respectively. Additionally, 20.8% of the patients presented with metastases at diagnosis, which reflects a pattern of advanced disease in Brazilian patients [5].

OS treatment involves combined high-dose chemotherapy and resection of the primary tumor and all metastatic disease sites usually via exploratory thoracotomy [6]. The pulmonary recurrence of the disease is frequent and associated with a poor prognosis and a post-relapse 5-year survival of 20% [7]. Thus, the detection of metastasis at diagnosis and the monitoring of metastatic lesions are essential in the treatment of OS. New strategies of treatment have been proposed by the BOTG to improve the survival of OS patients. However, no improvements in survival have been achieved even after the intensification of OS treatment [5].

Genetically, OSs are extremely complex tumors, and despite the knowledge about the molecular changes involved in the pathogenesis of OS [8], metastatic disease is not well understood in terms of the evolution of the disease. Recently, high levels of the expressions of the *MAPK7* and *MAP2K4* genes have been reported to be significantly associated with metastasis in OS [9].

In this context, long non-coding RNAs (lncRNA) are emerging as important molecular markers of metastatic disease in several human cancers [10–13]. LncRNAs are defined as endogenous RNAs larger than 200 nucleotides that do not contain significant open reading frames (i.e., no open reading frames greater than 100 amino acids) and thus do not result in proteins [14]. LncRNAs comprise a heterogeneous group of RNA molecules that is involved in the regulation of gene expression [15], protein localization [16] and the formation of essential protein complex substructures [17].

Hepatocellular carcinoma up-regulated long non-coding RNA (*HULC*) is an lncRNA that was first reported to be highly expressed in primary tumors of the liver [18]. Since this report, important roles of *HULC* have been described in the cellular invasion and metastatic processes of several human tumors [13,19,20].

Evaluations of lncRNA expression in OSs remain rare. Because metastatic disease is a challenge in OS, there is a critical need to identify markers that may indicate or accurately predict the existence of metastatic disease in OS patients. Therefore, we evaluated the expression of *HULC* in primary osteosarcoma samples at diagnosis and its association with the clinicopathological characteristics of OS Brazilian patients. We also evaluated the role of *HULC* expression as a potential prognostic marker in OS.

## Material and Methods

The study was conducted following national and institutional ethical policies, and it was previously approved by the Barretos Cancer Hospital Ethical Committee and the Medicine School of Ribeirão Preto/USP Ethical Committee (protocol CAAE 13671113.0.0000.5437). The ethical committees classified this study has having minimal risk, ensuring confidentiality, and not resulting in any clinical influences due to changes in treatment or genetic counseling for the participants and their families. For these reasons, both ethical committees waived the need for consent.

### Patient samples

Fresh-frozen osteosarcoma tissue samples from 33 patients were retrieved from the Biobank of the Barretos Cancer Hospital and the Department of Pediatrics at the Ribeirão Preto School of Medicine, University of São Paulo. All patients were diagnosed between 2006 and 2013, and the samples were taken from primary tumor biopsies prior the use of any possible systemic treatment and without metastasis at diagnosis. All patients were treated following the new clinical protocol of the Brazilian Osteosarcoma Treatment Group, which is currently referred to as the Latin American Group (GLATO) 2006. In the GLATO 2006 study, initially non-metastatic patients at diagnosis undergo the standard regimen of Cisplatin and Doxorubicin (60 mg/m²/day and 37.5 mg/m²/day, respectively; D1-D2; weeks 1 and 6) followed by Methotrexate (12 g/m²/day; D1; weeks 4–5 and 9–10). Surgery of primary tumor is proposed in week 11–12. After surgery, patients were randomized into a first arm treatment comprising four more cycles of chemotherapy, as described above, for a total of 31 weeks of treatment; and, in a second arm, in which after 31 weeks (same as first arm) Cyclophosphamide (25 mg/m²/day; every day) and Methotrexate (1.5 mg/m²/2x day; D1 and D4, every week) were administered for a total of 104 weeks of treatment.

### RNA extraction and cDNA synthesis

The OS samples were re-evaluated by a pathologist to confirm the diagnoses and to guarantee at least 80% tumor cellularity prior to RNA extraction. Total RNA was extracted from the OS samples using a standard protocol with TRIZOL reagent (Invitrogen, Grand Island, NY, USA). The homogenization of the tumor cells in TRIZOL was performed using Precellys 24 (Bertin Technologies, Rockville, MD, USA). RNA was quantified using Qubit 2.0 (Life Technologies, Grand Island, NY, USA), and quality was assessed using a 2100 Bioanalyzer (Agilent Technologies, Santa Clara, CA, USA). We considered samples with a RIN ≥ 4 for our analysis.

The RNA was submitted to treatment with deoxyribonuclease I (Sigma-Aldrich, St. Louis, MO, USA) following the manufacturer’s recommendations. cDNA synthesis was performed with 1 μg of DNA-free total RNA using the High-Capacity cDNA Reverse Transcription Kit (Life Technologies, Grand Island, NY, USA) according to the manufacturer's recommendations.

### Real-time quantitative PCR analysis of gene expression

The expression analysis of *HULC* was performed by RT-qPCR using PrimeTime qPCR assays (Integrated DNA Technologies, Coralville, Iowa, USA). PrimeTime assays contain probes and primers that allow for the specific amplification and evaluation of the expression of the gene of interest. For *HULC*, we used assay Hs.PT.58.3962267.

RT-qPCR was performed with 1 μL of the cDNA, 1x Master Mix Fast Probe KAPA (Kapa Biosystems, Wilmington, MA, USA), and 1x PrimeTime qPCR assay (50X) in a final volume of 20 μL. The reaction was performed as follows: 95°C for 10 minutes; 40 cycles at 95°C for 15 seconds; and 60°C for 1 minute. Additionally, we evaluated the expressions of the endogenous genes *GAPDH* and *ACTB* to normalize the expression data.

The normalized expressions of the gene of interest were calculated with the 2^-ΔCt^ mathematical model wherein ΔCt corresponds to the Ct of the gene of interest subtracted from the Ct of the endogenous gene [21]. The expression analysis was performed with the 7900 HT Fast Real-Time PCR System (Applied Biosystems, Grand Island, NY, USA), and all reactions were performed in duplicate.

### Clinical characteristics and statistical analysis

The epidemiological variables considered in the present study were the following: sex and age at diagnosis, and the clinicopathological variables of OS histological type, Huvos grading, tumor localization, tumor size and the occurrence of metastasis during treatment. The categorical variables are expressed as frequencies and percentages.

To initially categorize *HULC* expression, a cut-off value was determined using receiver operating characteristic (ROC) analysis. The cut-off value was determined as the value that maximized the sensitivity and specificity of the discrimination between non-metastatic patients and patients who experienced metastasis during treatment. Tumors with *HULC* expression levels below the cut-off were classified as low expression tumors, and those with *HULC* expression levels above the cut-off were classified as high expression. The sensitivity, specificity, positive predictive value, negative predictive value, and accuracy and the respective 95% confidence intervals were estimated. Furthermore, the gene expression levels (i.e., low or high expression) were correlated with the clinicopathological characteristics using the chi-square test or Fisher's exact test.

Overall survival and event-free survival were determined with the Kaplan-Meier method. For overall survival, death from any cause was considered an event, and the follow-up was determined from the date of diagnosis to the date of death. For event-free survival, the occurrence of metastasis during treatment and death were considered events, and the follow-up was determined from the date of diagnosis to the date of the event that occurred first. The differences between the survival curves were analyzed using the log-rank test.

For all analyses, statistical significance was considered at p<0.05. The statistical analyses were performed with SPSS v.21 software.

## Results

### Associations between *HULC* expression and the clinicopathological characteristics of the OS patients

The clinicopathological characteristics of the OS population are summarized in Table 1. We determined a *HULC* expression cut-off value to discriminate the patients with good outcomes (no event occurrences) from the patients with poor outcomes (occurrence of any event during the follow-up) using ROC analysis (Table 2). Based on this cut-off value, the *HULC* expressions were classified as low or high for the subsequent analysis.

**Table 1:** Clinicopathological characteristics of the osteosarcoma population.

Clinicopathological characteristics		N	%
Age at diagnosis (years)	<10	06	18.2
	≥10	27	81.8
	mean 15		
Gender	female	17	51.5
	male	16	48.5
Tumor site	femur	12	36.4
	tibia	12	36.4
	humerus	06	18.2
	fibula	03	9.0
Histological type*	osteoblastic	24	77.4
	chondroblastic	03	9.7
	fibroblastic	03	9.7
	telangiectatic	01	3.2
Huvos grade*	grade 1	07	23.3
	grade 2	14	46.7
	grade 3	07	23.3
	grade 4	02	6.7
Tumor size* (cm)	<12	11	52.4
	≥12	10	47.6
Metastasis (during treatment)	no	23	69.7
	yes	10	30.3

*Number of patients with available information.

**Table 2:** *HULC* expression cut-off based on predictive probability of clinical outcome among the OS patients.

Gene	Cut-off*	Sensitivity %	Specificity %	PPV %	NPV %	Accuracy %	AUC
		(CI)	(CI)	(CI)	(CI)	(CI)	(CI)
*HULC*	0.000048	50.0	73.7	58.3	66.7	64.6	0.48
		(23.0–76.9)	(48.8–90.8)	(27.7–84.8)	(43.0–85.4)	(50.4–76.5)	(0.25–0.70)

*The optimal cutoff was determined as the point at which it simultaneously maximized sensitivity and specificity

**PPV:** positive predictive value; **NPV:** negative predictive value. **CI:** 95% confidence interval.

We observed that *HULC* expression was not significantly associated with the evaluated clinicopathological characteristics of the osteosarcoma patients (Table 3).

**Table 3:** Associations between *HULC* expression and clinicopathological characteristics of the OS patients.

		*HULC* expression			
		Low expression		High expression		
		N	%	N	%	p-value
Age at diagnosis (years)	< 10	06	28.6	0	0.0	0.065
	≥ 10	15	71.4	12	100.0	
Gender	female	11	52.4	06	50.0	0.999
	male	10	47.6	06	50.0	
Tumor site	Femur	06	28.6	06	50.0	0.274
	Other	15	71.4	06	50.0	
Histological type	Osteoblastic	13	68.4	11	91.7	0.201
	Other	06	31.6	01	8.3	
Huvos grade	Grade 1/2	13	72.2	08	66.7	0.999
	Grade 3/4	05	27.8	04	33.3	
Tumor size (cm)	<12	07	58.3	04	44.4	0.670
	≥12	05	41.7	05	55.6	
Metastasis (during treatment)	no	15	71.4	08	66.7	0.999
	yes	06	28.6	04	33.3	

### Association of *HULC* expression with the prognoses of the osteosarcoma patients

**Fig 1:**
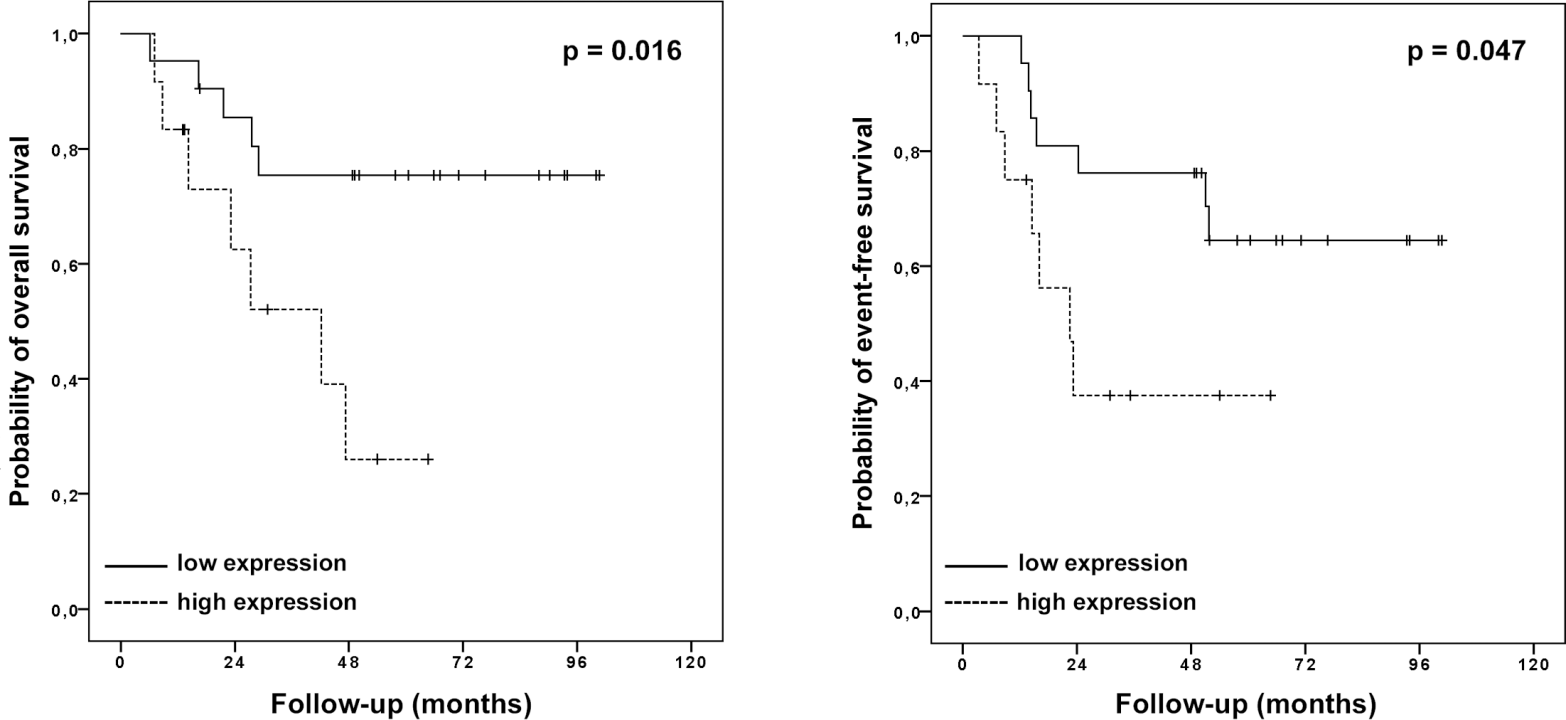
Kaplan-Meier survival curves of the OS patients according to *HULC* expression. A: overall survival, B: event-free survival.

The patients with higher *HULC* expression presented 5-year overall survival rate of 26.0% compared with 75.4% for those with low expression (p = 0.016). Similar results were observed for the EFS; i.e., the patients with high *HULC* expression presented a 5-year EFS rate of 37.5% compared with 64.5% for those with low expression (p = 0.047; Fig 1).

Regarding the evaluated clinical characteristics, our data revealed that the histological type of OS significantly influenced the EFS rate. The patients with the osteoblastic subtype presented a 5-year EFS of 69.8% compared with 21.4 for the other OS histologies (p = 0.047).

The COX regression analysis of the EFS revealed that *HULC* expression and histological type were independent prognostic factors for the OS patients (Table 4). These findings suggest that *HULC* expression is a biomarker of poor prognoses in OS patients and highlight the role of *HULC* in the biology of the disease.

**Table 4:** COX regression model of the event-free survival for the OS patients.

Variable	Category	Hazard ratio	95% CI (for Exp β)	p-value*
Histological type	Osteoblastic	1	-	-
	Other	22.78	2.32–223.97	0.007
*HULC* expression	Low	1		
	High	22.01	2.26–216.13	0.008

* Bold values are statistically significant (p<0.05)

## Discussion

Recent estimates suggest that there are more than 20,000 lncRNA transcripts in the human genome, which implies that the lncRNA class represents a large but unknown component of normal cellular networks that may be altered in tumor cells. Therefore, it is reasonable to assume that lncRNA deregulation is involved in the development of many diseases, including cancer [22]. Indeed, lncRNAs are often expressed in tissue-specific patterns; thus, these molecules are attractive new therapeutic targets [23,24].

OS is a complex genetic disease and despite all of the progress achieved by basic and clinical research in recent years, the molecular genetic mechanisms involved in the pathogenesis of osteosarcoma are not yet clear. LncRNA expression has not been well explored in OS, and our knowledge of how lncRNAs act in OS cells, particularly in terms of driving metastatic processes, remains very limited. Because the presence of metastatic disease at diagnosis drives OS patients to more aggressive treatment approaches [5], the identification of patients with non-metastatic statuses at diagnosis who will develop metastatic lesions during treatment is of significant interest. The identification of potential metastatic OSs at diagnosis even when clinical metastasis is not present based on results from standard imaging techniques could be a promising tool in the management of OS patients. In our work, we evaluated the expression of *HULC*, which is a lncRNA that is associated with the metastatic processes of tumors [13,18–20] in OS pediatric primary samples from patients without metastatic disease at diagnosis.

Initially, *HULC* was described as a liver-specific non-coding RNA that is highly expressed in primary tumors of the liver [18]. Subsequently, high levels of *HULC* were detected in the plasma of hepatocellular carcinoma patients and found to be associated with tumor aggressiveness and progression. Based on these data, some authors have suggested that *HULC* is a potential prognostic biomarker in liver cancer [19]. Moreover, high levels of *HULC* expression have been described in liver metastases in colorectal carcinoma patients [25], and its expression has been associated with lymph node metastasis and vascular infiltration in pancreatic cancer [13] and advanced metastatic disease in gastric cancer [20].

*HULC* has been demonstrated to act as a microRNA (miRNA) sponge that binds to and reduces the expression of a number of miRNAs, including miR-372. The reduction of miR-372 expression leads to increased expression of its target PRKACB, which in turn induces CREB phosphorylation. This process results in alterations in the patterns of the deacetylation and methylation of histones, which leads to chromatin remodeling and influences the expression of a series of genes [26].

We found no significant associations between *HULC* expression and the clinicopathological characteristics of the OS patients, which was probably due to our limited sample. Due to the rarity of this disease, the majority of the studies that have aimed to examine the molecular changes that occur in osteosarcomas have encountered restricted sample sizes [9,27,28]. Although, higher expression of *HULC* is not significantly associated with development of metastasis during treatment in our work, probably due to the number of patients (10) included in this group, our data demonstrated that elevated *HULC* expression is associated with poor prognoses among OS patients and influences both overall and event-free survival. Additionally, our observations regarding EFS, in which we considered the occurrence of metastasis during treatment and death as events, allow us to suggest that the high expression of *HULC* could be associated with primary tumors with a more aggressive behavior and/or metastatic potential.

Recently, elevated expression of *HULC* was associated with clinical stage and distant metastasis in OS. Additionally, elevated *HULC* expression has been associated with poorer overall survival in these patients [29], which corroborates our findings. Moreover, these authors demonstrated that the inhibition of *HULC* using a siRNA technique results in decreases in the proliferation, migration and invasion capacities of OS cell lines [29]. The results obtained in our work, considering a Brazilian OS population with a different genetic background from previous study [29] supports that in general *HULC* is a potential molecular marker of prognosis in OS. Also, our findings strengthen the observation that *HULC* seems to have a pivotal role in OS tumorigenesis.

In conclusion, our data suggest that *HULC* expression is associated with poor clinical outcomes in OS patients and that *HULC* could be a potential biomarker of the prognosis and occurrence of metastasis in OS. Further evaluations of larger samples are needed to allow for an extensive exploration of the role of *HULC* in osteosarcoma biology.
